# Anomalous Extensor Indicis With an Intermediate Tendon: Surgical and Functional Considerations

**DOI:** 10.7759/cureus.110229

**Published:** 2026-06-04

**Authors:** Nidhi Sunhare, Padamjeet Panchal

**Affiliations:** 1 Anatomy, All India Institute of Medical Sciences Patna, Patna, IND

**Keywords:** anatomical variation, cadaveric dissection, dorsal digital extensor expansion, dorsal wrist capsule, double-bellied muscle, extensor indicis proprius, extensor retinaculum, fourth extensor compartment, tendon transfer, tenosynovitis

## Abstract

The extensor indicis proprius (EIP) is regarded as one of the most consistent muscles of the dorsal forearm, originating from the distal third of the posterior ulnar surface and passing through the fourth extensor compartment to insert into the dorsal digital extensor expansion (DDEE) of the index finger. Despite its relative constancy, the EIP exhibits a notable range of morphological variations, including supernumerary tendons, anomalous insertions, and accessory muscle bellies. A double-bellied configuration of the EIP, particularly with an intermediate tendon bearing a capsular attachment, represents an exceptionally rare anatomical variant with significant biomechanical and clinical implications.

During routine cadaveric dissection of a formaldehyde-fixed female cadaver (approximately 55 years of age), an unusual double-bellied EIP with an intermediate tendon was identified. The proximal belly followed a typical course, transitioning into a tendon that traversed beneath the extensor retinaculum. Distal to the retinaculum, this intermediate tendon gave rise to a second, distinct fleshy belly extending approximately 3.5 cm over the dorsum of the hand before resuming a tendinous form and inserting normally into the DDEE of the index finger. The intermediate tendinous segment also gained an attachment to the dorsal capsule of the wrist joint near the third metacarpal. The extensor digitorum communis (EDC) slip to the index finger demonstrated a normal insertion pattern, and no supernumerary EIP tendons were identified. This variant is interpreted in the context of embryological, evolutionary, and functional frameworks. Incomplete regression of the distal premuscle mass during development likely underlies the persistence of a secondary distal belly, while the capsular attachment of the intermediate tendon may reflect retained embryonic fascial connections analogous to capsular muscles in other joints, such as the popliteus. Functionally, the additional contractile element may augment force distribution and fine motor control of index finger extension, though compartmental crowding within the fourth extensor compartment raises the risk of tenosynovitis, extensor lag, and localized dorsal hand pain. From a surgical standpoint, this variation is directly relevant to tendon transfer procedures, reconstructive hand surgery, and decompression of the fourth dorsal compartment, where failure to recognize such anatomy may lead to inadvertent tendon injury or suboptimal outcomes.

This case expands the documented morphological spectrum of EIP variants and elaborates the importance of recognizing double-bellied configurations with intermediate capsular attachments. Surgeons and anatomists should remain vigilant for such anomalies during preoperative planning and cadaveric dissection, particularly in the context of tendon harvest, transfer, or dorsal wrist decompression procedures.

## Introduction

The extensor indicis proprius (EIP) is among the muscles of the deep group of the posterior compartment of the forearm. It originates from the lower one-third part of the ulnar posterior surface along with the interosseous membrane [1], just after the attachment of the extensor pollicis longus (EPL). The fleshy, muscular belly of EIP lies on the medial aspect of the EPL. It lies deep to the extensor digitorum communis (EDC), usually passes as a single tendon [2] on the dorsal surface of the distal ulna through the fourth extensor compartment of the wrist [3], along with the index finger slip tendon of EDC, enclosed in a common tendinous sheath beneath the extensor retinaculum. The muscle typically inserts into the dorsal digital extensor expansion (DDEE) of the index finger, specifically on the medial side of the extensor digitorum tendon [4]. The radial nerve, via its posterior interosseous branch (C7, C8), innervates the EIP muscle, while the posterior interosseous artery maintains the blood supply. Functionally, EIP independently extends the index finger and assists in wrist extension. The first dorsal and palmar interossei and first lumbrical muscles assist EIP in the index finger interphalangeal joint extension [5]. Its role in fine motor control and precision grip is particularly significant, as it contributes to hand dexterity and the ability to perform intricate tasks. The extensor digitorum brevis manus (EDBM) is a small, rare accessory muscle on the dorsum of the hand, considered a normal anatomical variant. It arises from the dorsal wrist capsule or distal radius and inserts into the extensor expansion of the index or middle finger. Clinically, it may be mistaken for a ganglion or soft tissue swelling. Ogura’s classification describes three patterns of the EDBM. In Type I, the EIP is absent, and the EDBM is inserted on the index finger. In Type II, both EIP and EDBM insert on the index finger, with subtypes depending on whether the EIP is vestigial and merges with EDBM (IIa), the EDBM joins the EIP tendon (IIb), or the EDBM inserts separately on the ulnar side, sometimes with a slip to the middle finger (IIc). In Type III, the EIP is inserted normally on the index finger, but the EDBM is inserted on the middle finger, occasionally with an accessory EIP slip [6].

The extensors of the forearm are known to exhibit a wide range of anatomical variations, including differences in muscle bellies, tendon structure, and insertion patterns [7-12]. The EIP tendon typically inserts onto the ulnar border of the first digital slip of EDC attachment at the dorsal aspect of the second metacarpophalangeal joint, thereby earning the designation EIP. However, when the EIP tendon inserts onto the middle finger, it is termed the extensor medii proprius or digiti (EMP) [8,13]. In some cases, the EIP tendon may be duplicated, with both tendons inserting onto the index finger (EIP) or one attaching to the index finger while the other inserts onto the middle finger, referred to as the extensor indicis et medii communis [13]. Additionally, a rare variation exists in which one tendon inserts onto the index finger and the other onto the thumb, termed the extensor pollicis et indicis (EPI) [7]. Exceptionally, the EIP tendon may give rise to three slips, with the third inserting onto the ring finger [13]. Yoon et al. intraoperatively identified the EIP deep to the EDC tendons on the dorsal hand, with its muscle belly extending anomalously distal to the extensor retinaculum across the metacarpal region. The myotendinous junction was located 5.7 cm distal to Lister's tubercle, and the tendon measured 5 cm from this point to the second metacarpal neck [14].

Anomalies of the EIP are not mere anatomical curiosities but have significant clinical implications. Variants may mimic ganglionic swellings on the dorsum of the hand [15], complicate surgical approaches, or interfere with tendon transfer procedures in which the EIP is often harvested [16,10]. Moreover, such anomalies shed light on the evolutionary and embryological development of the extensor musculature of the forearm and hand, reflecting the persistence of phylogenetic patterns observed in non-human primates [17-19].

## Case presentation

Both forearms and hands of a formaldehyde-fixed female cadaver, approximately 55 years old, were dissected in the Department of Anatomy during routine undergraduate dissection classes. A longitudinal incision was made along the dorsal aspect of the forearm and hand, with additional transverse incisions at the proximal and distal ends to enhance exposure. The skin flaps were then carefully reflected laterally and medially to preserve the underlying structures. On the dorsum of the hand, after dissection of the superficial fascia, the extensor retinaculum was divided longitudinally to reveal the tendons. The extensor tendons were identified within their respective compartments, and their arrangement, along with any anatomical variations, was carefully noted. The EIP and EDC, along with all anatomical variants and unusual tendons, were carefully examined and documented. Specifically, for the EIP tendon to each second digit, the number of slips at the origin, shape of the muscular belly, its relation in the extensor compartment, its relation to EDC, any extra tendons, additional muscular bellies, and insertion were meticulously recorded.

**Figure 1:**
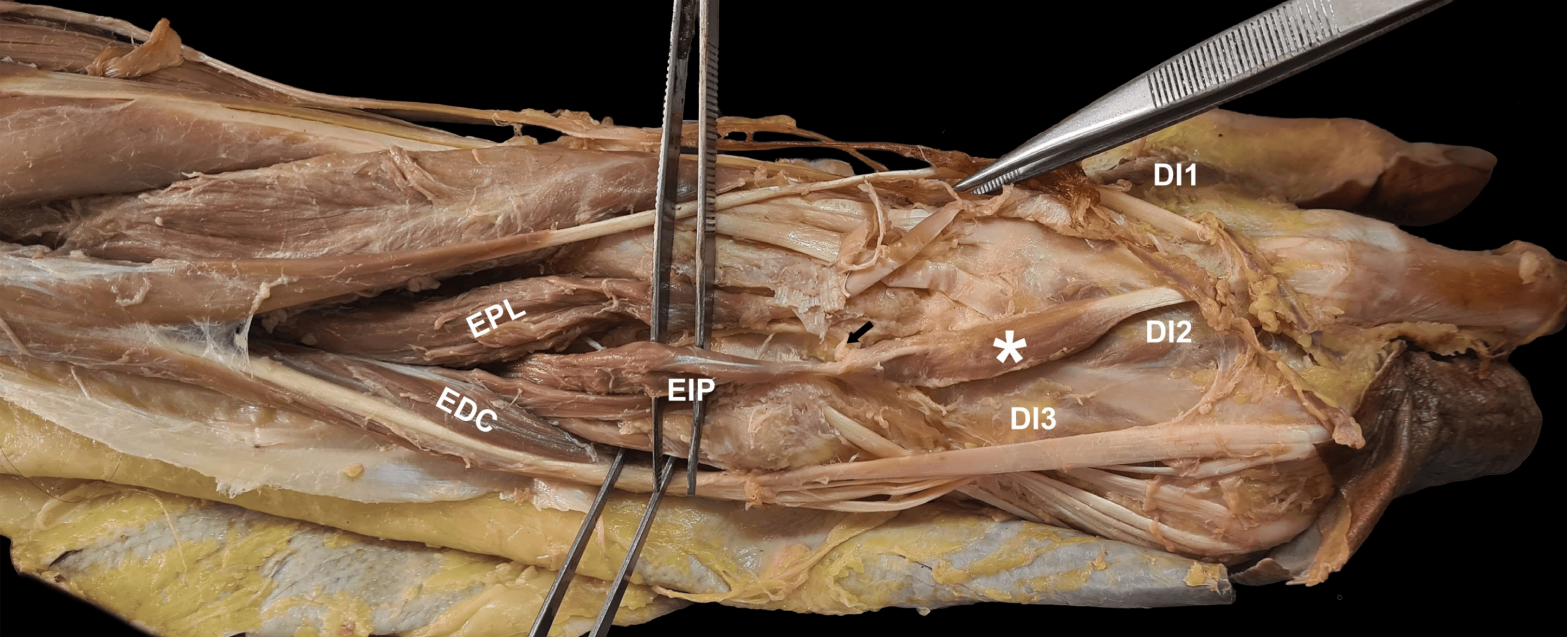
The right posterior forearm, wrist, and dorsum of the hand showing the extensor indicis proprius (a proximal muscle belly arising from the lateral surface of the distal ulna and a distal belly connected by an intermediate tendon). (*) denotes the distal belly of extensor indicis proprius. A small black arrow shows the area of attachment of the intermediate tendon to the capsule of the wrist joint. EPL: extensor pollicis longus; EDC: extensor digitorum communis; DI: dorsal interossei; EIP: extensor indicis proprius

The right and left side EIP muscles originated from the distal third of the lateral aspect of the ulna and the adjacent interosseous membrane. Both presented as a single muscle belly tapering into a single tendon. The fleshy belly of the EIP was innervated by the posterior interosseous nerve. Careful morphological examination of the site of origin revealed no additional slips. Distally, the muscle belly continued as a single tendon. The tendon had its typical course through the fourth compartment of the extensor retinaculum. Beyond this point, however, the tendon unexpectedly returned to a fleshy belly on the right side, compared with the left. Later, the tendon again turns into a fleshy belly, which extends approximately 3.5 cm distal to the extensor retinaculum on the dorsum of the hand and again becomes tendinous to insert into the DDEE of the right index finger (Figure 1).

**Figure 2:**
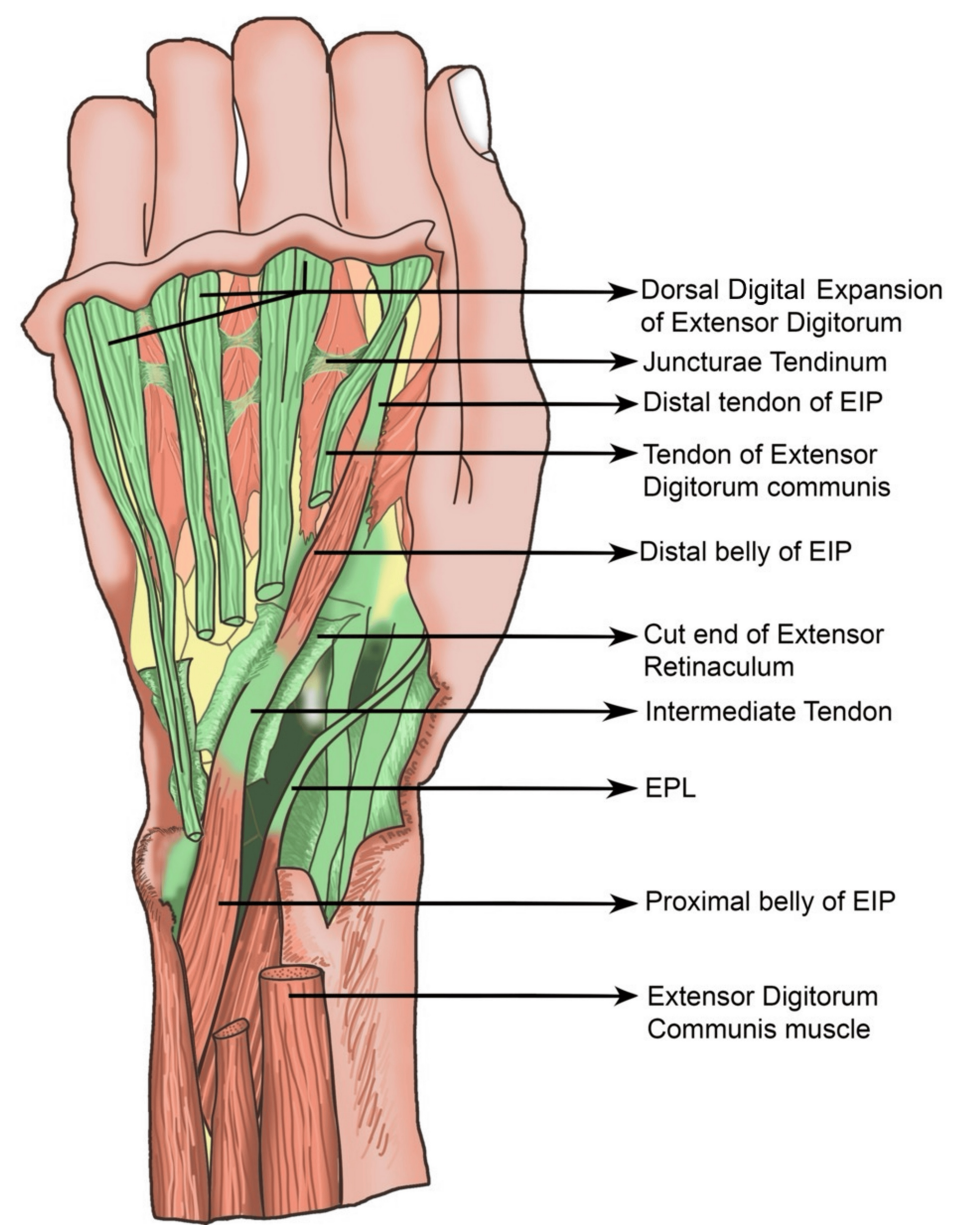
Schematic diagram of the dorsum of the hand showing the EIP, proximal and distal bellies with the intermediate tendon after opening the compartment of the extensor retinaculum. The small intermediate tendon passes through the fourth compartment of the extensor retinaculum. The tendinous slip of the EDC has a normal attachment in the index finger. Illustration created by Mr. Menajuddin Ansari (Artist in the Department of Anatomy). The illustration was created using Adobe Photoshop 2026 (Adobe Systems Software Ireland Ltd., Dublin, Ireland). EPL: extensor pollicis longus; EIP: extensor indicis proprius; EDC: extensor digitorum communis

An intermediate tendinous part may serve an accessory function. After passing through the extensor retinaculum, the intermediate tendon gains an attachment to the capsule on the dorsal aspect of the wrist joint near the dorsal aspect of the third metacarpal. The tendinous slip of the extensor digitorum for the index finger was observed to have a normal insertion (Figure 2).

## Discussion

The EIP is considered one of the most constant muscles of the dorsal forearm, yet literature demonstrates significant variability [7,8]. Supernumerary tendons, anomalous insertions, and bifid or duplicated bellies have been reported in diverse populations [12,15]. In the present study, no supernumerary tendons of the EIP were observed. The present case of a double-bellied EIP with an intermediate tendon anchored to the dorsal wrist capsule expands the known spectrum of such anomalies. The double-bellied EIP is an uncommon anatomical variation that has significant biomechanical implications. This variation, with its two distinct muscle bellies, can affect the muscle's functional modality and the biomechanics of the hand. 

Anatomical series consistently demonstrate that while EIP exists almost invariably, its morphology and insertion are not uniform [10,16,17]. Variations include accessory slips to the middle or ring fingers, bifurcation of the tendon, or duplication of the muscle belly. The presence of a distal belly on the dorsum of the hand, as observed here, challenges the conventional description of the EIP being proximally confined and reinforces the dynamic adaptability of this muscle [18].

Embryological basis

Embryological evidence supports the case report's findings. The deep extensors of the forearm, including the EIP, originate from a distal premuscle mass that migrates proximally during development [19]. Incomplete regression or persistence of segmentation within this mass may result in accessory slips, bifid bellies, or connections to adjacent fascial planes [20]. The attachment of the intermediate tendon to the dorsal wrist capsule observed in this case likely reflects the persistence of such embryonic connections.

Phylogenetic perspective

From an evolutionary perspective, these anomalies represent vestiges of a phylogenetic stage when primates retained numerous independent extensors, reflecting their need for grasping and climbing [7,21,22]. In humans, reduction and fusion of these muscles have simplified the extensor anatomy; however, independent index finger extension has been preserved as a functional necessity. Morphological variations in the EIP across human populations may therefore reflect residual phylogenetic plasticity, providing insight into the evolutionary basis of fine motor control [12,15,21].

Functional and biomechanical implications

Functionally, a double-bellied EIP has important biomechanical consequences. A second distal belly creates an additional contractile element that may enhance force distribution and fine control of index finger extension [23]. The fascial connection of the intermediate tendon to the dorsal wrist capsule could also contribute to the dynamic stabilization of the joint. However, such modifications may predispose to compartmental crowding within the fourth extensor compartment, potentially resulting in tenosynovitis, extensor lag, or localized pain [17].

Clinically, patients may present with either painful or painless swelling on the dorsal aspect of the hand. In some cases, the swelling is associated with radiating pain into the index finger or distal forearm, while in others, it remains localized without any radiating symptoms. In some cases, surgical exploration has revealed synovitis as the cause of pain and an abnormally elongated distal muscle belly of the EIP within the fourth dorsal compartment. The common differential diagnoses include trauma, tenosynovial disease, ganglion cysts, and soft tissue tumors. However, pain due to EIP muscle pathology is rare [10,15,24].

Ogura and colleagues performed a comprehensive anatomical investigation of 559 dissected cadavers and proposed that the EDBM muscle is a variation of the EIP muscle, as both share a common neurovascular bundle. The tendinous attachment of EIP on the wrist joint capsule suggests a possible proprioceptive role in wrist stability. This adaptation is functionally similar to capsular muscles found in other joints, such as the popliteus in the knee, which contributes to joint stabilization. The accessory muscle corresponding to the EDBM was found, inserting into the EIP tendon [6].

Cauldwell et al. reported that the musculotendinous junction of the EIP was located within the fourth dorsal compartment in 75% of specimens, while in 4% of cases, it extended distal to the extensor retinaculum. They found three cases with an abnormal muscular origin of EIP among 263 cases. In one case, they discovered that the EIP muscle originated typically from the ulna, transitioned into a tendinous form, and then regained a muscular structure before making a secondary attachment near the carpal bones [7].

Clinical significance

There is evidence suggesting that the EPL and EIP muscles phylogenetically evolved from the EPI, which originated from a common ancestral muscle [8]. The common origin of the EIP and EMP, along with the presence of the extensor indicis et medii communis (EIMC), supports the notion of a single embryologic origin for these muscles [13]. Yoshida reported 11 cases out of 832 in which the EPL and EIP were fused, resulting in the absence of a distinct separation between the two muscles [12].

The EI tendon is commonly used in tendon reconstruction procedures to restore function in other digits, highlighting the need to anticipate such variations and conduct pre-operative evaluations to confirm its presence when planning these procedures. Clinically, the EI plays a crucial role in tendon transfer surgeries, particularly in cases involving hand injuries, nerve palsies, or reconstructive procedures to restore independent extension of the index finger. Surgeons may utilize the accessory belly of the EIP muscle as a graft. The occurrence of such uncommon anatomical variation, such as a double-bellied EI or an intermediate tendon attaching to the wrist capsule, may influence surgical approaches. It can pose procedural difficulties due to altered typical anatomy, making dissection more complex during surgical interventions and affecting clinical outcomes. There is a high chance of inadvertent tendon injury. Understanding these anomalies aids in precise surgical planning, helping to minimize complications and optimize functional outcomes, especially in tendon transfers and reconstructive procedures [4].

The EI variations can influence muscle function, biomechanics, and surgical approaches, making their recognition essential in clinical and anatomical studies [20]. A clinical test described by Spinner and Olshansky is utilized to confirm the diagnosis of EIP syndrome [25]. The EIP possesses an anomalously distal muscle belly that extends into the fourth dorsal compartment beneath the extensor retinaculum. During forceful or repetitive wrist activity, this muscle belly becomes compressed beneath the extensor retinaculum, resulting in the characteristic painful swelling [26]. Pre-operative imaging, including MRI or ultrasound, is advisable to delineate variant anatomy prior to surgical intervention [3]. If a compressive syndrome is suspected, resection of the anomalous muscle belly is recommended [24]. 

## Conclusions

The present case documents an uncommon anatomical variation of the EIP muscle, characterized by a double-bellied configuration with an intermediate tendon anchoring to the dorsal wrist capsule. This variation expands the known morphological spectrum of the EIP and emphasizes the inherent variability of the extensor musculature of the forearm and hand, despite the EIP being regarded as one of its most constant components. Functionally, the additional distal muscle belly may enhance force distribution and contribute to dynamic wrist stabilization. However, it may also predispose to compartmental crowding, tenosynovitis, or extensor lag. Awareness of such variations is paramount for the accurate differential diagnosis of dorsal hand swellings, meticulous surgical planning, and the safe execution of tendon transfer procedures in which the EIP is routinely utilized as a donor tendon.
